# The Sanitary Labours of Hector Gavin, M.D.

**Published:** 1855-06

**Authors:** 


### THE SANITARY LABOURS OF HECTOR GAVIN, M.D.

THE death of our distinguished friend, Dr. Gavin, from its suddenness, the unhappy circumstances under which it occurred, and the prominent position which he held at the time as one of the health officers of the Crimean expedition, naturally gave rise to much kindly feeling and regret. It has been suggested to us by many friends, that a brief outline of the sanitary labours of one who once held the same public position as ourselves, namely, that of editor of a journal devoted to public health topics, might very fittingly be placed on permanent record in these pages.

This suggestion we act upon with sincere pleasure, regretting only the necessity of a task so sorrowful. We have no details of the career of Dr. Gavin previous to the period when his medical education was completed. He took his degree as doctor of medicine in the University of Edinburgh in the year 1836, having signalised himself remarkably during his student life, as one possessing great energy of character and love of his profession. He was elected president of the Hunterian Society during this period; and had the honour of presiding over great numbers of the active student members of the university, some of whom have since risen to great estimation.

In the session of 1835, Sir George Ballingall, the distinguished professor of military surgery, offered a prize to the students of the university for the best essay "On the Classification of the Feigned and Factitious Diseases of Soldiers and Sailors, on the means used to simulate or produce them, and on the modes of detecting Impostors." Dr. Gavin was the successful competitor; and so highly was his essay thought of by Sir James Macgrigor, the director-general of the medical department of the army, that he advised the author to extend the work, so that he might recommend it as one of the books with which medical officers of the army should provide themselves upon entering the service. This rare honour is sufficient testimony of the great merits of the essay. Sir William Burnett also expressed his high opinion of its usefulness; and a similar compliment was paid by some of the leading medical officers in the East India Company's service.

This was a happy augury for Dr. Gavin at the opening of life; and to it may doubtless be traced much of the bold relief and colouring of his future career.

In 1838, Dr. Gavin, after having become a fellow of the Royal College of Surgeons of Edinburgh, established himself in London as a general practitioner of medicine in the Hackney Road, where he ever afterwards continued to reside. He was soon appointed surgeon of the London Orphan Asylum, and of the British Penitent Female Refuge.

From an early period in his professional career in London, his mind had been drawn to the subject of Preventive Medicine; and from his acquaintance with Dr. Southwood Smith, by whom from the first he was most highly esteemed, his attention was naturally directed into that channel of scientific inquiry, in which he was destined to play so conspicuous a part. With his inherent ardour of temperament, he threw himself heart and soul into the ranks, then very small, of the sanitary reformers, who laid the foundation of all the legislative measures which have of late years been enacted.

Soon after this, Dr. Gavin became one of the most active and hard-working members of the Health of Towns' Association, whose labours did so much to press the subject of sanitary reform on the attention of the public; and about the same time he commenced a series of lectures on "Forensic Medicine and Public Health" at the Charing Cross Hospital School of Medicine.

In 1848, he published his "Sanitary Ramblings," being sketches and illustrations of Bethnal Green, a "Type of the Condition of the Metropolis and other large towns";—a work which in itself is sufficient to testify to the disinterested devotedness and philanthrophy which animated the author in his zeal to promote the progress of what had now become the ruling passion of his professional life—the amelioration of the condition of the poor, and the prevention of disease and death.

In the summer of 1849, he became editor of the "Journal of Public Health and Record of Sanitary Improvement," and fulfilled the duties of the office till the publication was discontinued; and during the severe outbreak of cholera in the metropolis in that year, he received the first public recognition of his meritorious exertions. The General Board of Health called upon him to establish and carry out the necessary precautionary and preventive measures in several of the districts of the metropolis, which had been placed by the Board under the general superintendence of Mr. Grainger.

The excellent report drawn up by the latter gentleman contains large extracts from the evidence furnished by Dr. Gavin of the results of the house-to-house visitation in the parishes of Bethnal Green and Shoreditch. His exertions elicited the strongest approval of the General Board of Health.

As soon as the cholera pestilence subsided in the metropolis, and his public services were no longer required, he set about establishing the "Metropolitan Sanitary Association," which, under the presidency of the Bishop of London, aided in no small degree the movement in favour of sanitary legislation. Dr. Gavin, in conjunction with Dr. Burnett and the Rev. W. Lusignan, undertook the duties of the secretaryship, and zealously discharged his part. The first report of the Association, embracing the whole subject of sanitary reform, and to which Dr. Gavin largely contributed, was published in the summer of 1850.

At the close of that year, a new and most important field of usefulness was opened to him. In consequence of the dreadful ravages of cholera in Jamaica, and the apprehensions entertained in the other West India colonies of the visitation of the pestilence, government resolved to send out three sanitary commissioners to counsel and direct the local authorities as to the necessary measures for the immediate safety and permanent improvement of the colonies. Dr. Gavin was one of them, the other two being Dr. Milroy and Dr. Laidlaw. During the two years and a half that he remained abroad, he visited Barbadoes, Trinidad, St. Vincent, the Bahamas, and British Guiana, collecting information, drawing up reports, framing bills on the subject of the public health, and aiding the local authorities in the establishment of needful sanitary enactments. The flattering testimonials which he received from Sir William Colebrooke, Lord Harris, Sir Henry Barkly, and the governors of other colonies, bear the most lively testimony to his zeal and ability in the discharge of his important mission.

While in the West Indies, Dr. Gavin completed his essay on "The Habitations of the Industrial Classes," which was published for the benefit of the Society for Improving the Condition of the Labouring Classes, and obtained a very extensive circulation.

Shortly after he returned home in 1853, the fearful attack of cholera at Newcastle occurred; and Dr. Gavin's services were engaged by the General Board of Health to organise the measures then so urgently required to mitigate its ravages in that most unwholesome and long neglected town. In spite of no small amount of opposition and obloquy from some of the residents, the prompt and energetic steps he took, and the ability with which he carried them out, gained for him the approbation and esteem of almost all the leading medical men. A highly complimentary memorial, signed by nearly thirty of his professional brethren, was addressed to him upon leaving Newcastle; and no one, who has read Dr. Gavin's evidence given before the Royal Commission of Inquiry into the Newcastle Epidemic, can fail to join heartily in the commendation of his conduct.

He was subsequently employed by the General Board of Health in different parts of Scotland where the cholera had appeared, more especially at Glasgow and Dundee.

All the leisure Dr. Gavin could spare from his more active engagements was devoted to the preparation of his reports on the different West India colonies which he had visited.

On the re-appearance of cholera in London in 1854, he was appointed by the Postmaster-General, Viscount Canning, to act as medical superintendent of the entire staff of the General Post-Office establishment during the continuance of the epidemic in the metropolis. The following letters from the Secretary will best show how highly his services were appreciated upon that occasion.

"General Post Office, *2nd Dec.* 1854.

"Sir,—The Postmaster-General has had before him your Report of the 28th ult., on the Cholera and the preventive and remedial arrangements which have been instituted and superintended by you in connexion with this Department; and his Lordship desires me to say that the result of the measures above referred to is most satisfactory, and that the best thanks of the Department and of the Government are due to you for your exertions and success.

"I have to add that the arrangements made by you may now cease.

"I am, Sir, your obedient servant,

"Rowland Hill.

"H. Gavin, Esq., M.D., &c., &c."

"Circulation Department, *Nov.* 1854.

"Sir,—The severe ordeal of Cholera and Diarrhœa through which we have lately passed having now happily subsided, we cannot delay the expression of our most grateful acknowledgments for the indefatigable zeal and ability with which you have, during this long and calamitous visitation, so successfully contended against the attacks of those complaints; and we doubt not thereby, under Providence, the lives of many of our number have been preserved.

"We would also thank you not only for the uniform kindness and consideration you have shown in administering to the wants and relieving the sufferings of those attacked, but also for the friendly counsel and advice you have given to all who have applied to you, and by which a feeling of confidence has been established throughout the Department; and in no case, we believe, have those who have adopted the precautions you have suggested failed to enjoy comparative security and good health.

"This confidence has not only been a blessing to those in the Post Office Department, but has in many cases been extended to the minds of the relations and friends of those employed in this Department.

"In taking leave of you we would, therefore, beg you to accept our best wishes for your health and happiness, and that you may enjoy the rewards we feel due to you for your great skill and attention.

"We are, Sir, your very obedient Servants,

"W. Bokenham,

"*Comptroller of the Circulation Department,*

"And 923 other Officers and Employés.

"Hector Gavin, M.D., &c."

The disastrous losses among our brave troops in the East, from the neglect of proper sanitary as well as of other precautionary measures, had, as might have been expected, early attracted the attention of a man of Dr. Gavin's character. He, like other zealous students of hygienic medicine, had addressed the Government upon the advantage of a sanitary staff being attached to the army; but the advice was at first unheeded. Upon the accession to office of Lord Panmure, and when the public mind had become so much excited, he again urged the point, and this time with success. He was the first selected to form the Sanitary Commission which was sent out last February; and never did a public servant engage with more high and holy zeal on an important mission than he did upon this—alas! his last—enterprise.

The very day before he received the appointment from Lord Panmure, he had been, in consideration of his valuable services in the preceding autumn, named permanent medical officer (we believe) to the General Post Office.

At the same time, he had just sent in to the Colonial Office the first of his reports on the West India Colonies. This report is entirely devoted to Barbadoes, and is a work of the most elaborate and comprehensive character, abounding in information, which, it is to be hoped, will be made generally known by its publication at the expense of the Government.

The foregoing brief record of the labours of Dr. Gavin shows how much the friends of sanitary improvement have to deplore his untimely loss.

He possessed, in an eminent degree, the *perfervidum ingenium* of his countrymen.

His zeal and ability had secured to him the cordial friendship of all the leading sanitary reformers of note,—the Earl of Shaftesbury, the Earl of Carlisle, Marquis of Normanby, Lord Ebrington, the Bishop of London, Dr. Southwood Smith, Mr. Chadwick, &c.

Like all earnest and thorough labourers in the field of sanitary reform, his zeal in the cause increased, year by year, with his knowledge and practical experience of its magnitude and importance. Like them, too, he was ever deeply grieved at the apathy and indifference of our legislators; and would often express his indignant feelings at the slighted labours of his own profession (to which sanitary science owed its origin and chief progress), by placemen who have reaped the fruits of those professional labours.

The career of Dr. Gavin affords a most useful example and encouragement to young medical men who are starting in the race of professional rivalry, with no patrons or friends save those whom they by their own merits and zeal may acquire.

